# Control of Artifactual Variation in Reported Intersample Relatedness during Clinical Use of a Mycobacterium tuberculosis Sequencing Pipeline

**DOI:** 10.1128/JCM.00104-18

**Published:** 2018-07-26

**Authors:** David H. Wyllie, Nicholas Sanderson, Richard Myers, Tim Peto, Esther Robinson, Derrick W. Crook, E. Grace Smith, A. Sarah Walker

**Affiliations:** a Nuffield Department of Medicine, John Radcliffe Hospital, Oxford, United Kingdom; b Public Health England Academic Collaborating Centre, John Radcliffe Hospital, Oxford, United Kingdom; c The National Institute for Health Research Health Protection Research Unit in Healthcare Associated Infections and Antimicrobial Resistance at University of Oxford, Oxford, United Kingdom; d Department of Bioinformatics, Public Health England, London, United Kingdom; e Public Health England National Regional Mycobacteriology Laboratory North and Midlands, Heartlands Hospital, Birmingham, United Kingdom; University Hospital Münster

**Keywords:** artifact, Mycobacterium tuberculosis, reference mapping, relatedness, single nucleotide variation

## Abstract

Contact tracing requires reliable identification of closely related bacterial isolates. When we noticed the reporting of artifactual variation between Mycobacterium tuberculosis isolates during routine next-generation sequencing of Mycobacterium spp., we investigated its basis in 2,018 consecutive M. tuberculosis isolates. In the routine process used, clinical samples were decontaminated and inoculated into broth cultures; from positive broth cultures DNA was extracted and sequenced, reads were mapped, and consensus sequences were determined. We investigated the process of consensus sequence determination, which selects the most common nucleotide at each position. Having determined the high-quality read depth and depth of minor variants across 8,006 M. tuberculosis genomic regions, we quantified the relationship between the minor variant depth and the amount of nonmycobacterial bacterial DNA, which originates from commensal microbes killed during sample decontamination. In the presence of nonmycobacterial bacterial DNA, we found significant increases in minor variant frequencies, of more than 1.5-fold, in 242 regions covering 5.1% of the M. tuberculosis genome. Included within these were four high-variation regions strongly influenced by the amount of nonmycobacterial bacterial DNA. Excluding these four regions from pairwise distance comparisons reduced biologically implausible variation from 5.2% to 0% in an independent validation set derived from 226 individuals. Thus, we demonstrated an approach identifying critical genomic regions contributing to clinically relevant artifactual variation in bacterial similarity searches. The approach described monitors the outputs of the complex multistep laboratory and bioinformatics process, allows periodic process adjustments, and will have application to quality control of routine bacterial genomics.

## Abstract (precis)

Contact tracing requires reliable identification of closely related bacterial isolates. When we noticed the reporting of artifactual variation between Mycobacterium tuberculosis isolates during routine next-generation sequencing of Mycobacterium spp., we investigated its basis in 2,018 consecutive M. tuberculosis isolates.

## INTRODUCTION

Identifying closely related bacterial isolates is required for clinical and epidemiological purposes (1–3). Most published approaches using short-read next-generation sequencing (NGS) rely on mapping to a high-quality reference sequence followed by consensus base calling (1–8). A known problem with this approach concerns the existence in many bacterial genomes of “hard-to-map” regions which either are repeated within the genome or contain regions of low sequence complexity. High-confidence mapping of short reads to such regions is difficult or impossible, and so determining the consensus sequence of these regions is difficult. One approach to managing this problem is to identify these regions bioinformatically prior to mapping by analysis of sequence complexity (9) or from repetitiveness within the genome (10). Base calls within these prespecified regions are then ignored (“masking”) when assessing relatedness of isolate sequences. A second complementary approach filters base calls based on read mapping confidence as reported by various mappers (11–14) in the form of mapping quality (MAQ) scores.

Mycobacterium tuberculosis is one of the most important pathogens of humans, with about 3 million cases of tuberculosis (TB) confirmed by culture globally each year (15). Recently, laboratory protocols have been described and deployed by Public Health England (4) in which the species and drug resistance of mycobacteria, including M. tuberculosis, are identified by sequencing microbial DNA. Laboratory processing of clinical samples suspected of containing mycobacteria involves decontamination using chemicals which kill nonmycobacterial species before the samples are inoculated into broth culture (16). Mycobacterial growth indicator tubes (MGITs) and associated tube monitoring equipment are a commercially available implementation of such a broth culture system.

In the process adopted by Public Health England, sequencing and bioinformatics analysis of DNA extracted from positive MGITs allows determination of mycobacterial species and drug resistance (17). This laboratory and bioinformatics process also allows the genetic distance between M. tuberculosis isolates to be estimated, using sequences derived by consensus base calling from mapped data. The organism coevolved with human populations as they migrated, generating multiple lineages which differ from the ancestral sequence by hundreds or thousands of single nucleotide polymorphisms (SNPs) (18), as well as small indels, gene deletions, and inversions (19). However, the evolutionary clock rate of the organism is slow, at about 0.5 single nucleotide variant (SNV)/genome/annum (3, 5, 7), and small numbers of SNVs are of clinical significance: studies based on retrospective collections of M. tuberculosis organisms grown on solid media prior to sequencing have proposed a threshold of 5 SNVs as compatible with recent transmission (3, 5, 7). The bioinformatics processes used for relatedness estimation in the deployed pipeline were also optimized using samples regrown from frozen stocks on solid media (Lowenstein-Jensen slopes) (16).

The quality of complex processes deployed in medical laboratories is ensured by adherence to quality standards, such as those laid out in ISO15189:2012 (20). These standards require that the processes followed and the environments in which they operate comply with patterns of work known to enhance the consistency and interpretability of the laboratory outputs. For example, in a drug testing laboratory, a set of samples of known composition may be run through the analyzers to confirm that particular commonly found substances which might potentially interfere with the assay (such as caffeine and paracetamol) have no impact on detection of the drug of interest. M. tuberculosis infection is commonly diagnosed from sputum samples, which contain a wide variety of organisms other than mycobacteria (21). DNA from such organisms may contain sequences homologous with those present in mycobacteria, for example, in highly conserved core bacterial genes. Therefore, this nonmycobacterial DNA has the potential to interfere with assays based on mapping of mycobacterial reference genome mapping.

In this study, we investigated the concept of interfering substances in the context of the detection of closely related M. tuberculosis isolates. In particular, we considered whether DNA of nonmycobacterial origin might cause interference. We describe a process which we call adaptive masking. This defines hard-to-map regions existing in the context of the laboratory, sequencing, and mapping processes being used, independently of predictions based on the reference sequence and of filtering based on reported mapping quality. Our work was motivated in part by observations from analysis of prospective sequencing of M. tuberculosis sequences in England using a previously described bioinformatics pipeline (17). It appeared that large SNV distances were being reported between isolates with a strong epidemiological likelihood of having recently transmitted to each (e.g., isolates with unusual resistance profiles from individuals who were cohabiting): that is, false-positive variation between isolates was being reported. We assess the impact of adaptive masking on addressing this problem and discuss quality control of relatedness monitoring in the context of continuous process monitoring in accredited clinical laboratories.

## MATERIALS AND METHODS

### Isolation of DNA from mycobacteria and sequencing.

Clinical specimens were decontaminated and inoculated into mycobacterial growth indicator tubes (MGITs) (16). Positive samples were extracted (4). After DNA extraction, Illumina sequencing libraries were prepared using either 11 (early in the study) or 15 (later in the study) mycobacterial DNA extracts, as previously described (4). All samples sent from patients for processing for mycobacteria between 1 May 2016 and 30 May 2017 to a single reference laboratory were studied; the catchment of this laboratory is approximately 15 million people, or about one-third the population of England.

### Bioinformatics processing.

Reads obtained from the MiSeq instrument were first examined for the presence of Mycobacterium tuberculosis using the Mykrobe tool, which detects species-specific k-mers (22). Only samples identified as being derived from the M. tuberculosis complex by Mykrobe (22) were considered in this work. Additional read classification was performed with Kraken (23), which assigned reads to bespoke database constructed from (i) all bacterial genomes deposited in the NCBI RefSeq database as of January 2017 and (ii) Genome Reference Consortium Human Build 38 (GRCh38) to allow detection of host DNA as described previously (24), but with k-mer reduction to 25 Gb. We quantified reads mapped to M. tuberculosis (NCBI taxonomy identifier: 77643), to nonmycobacterial bacterial species, and to humans. After this, human reads were discarded.

Reads were mapped to the H37Rv v2 genome (GenBank accession number NC_000962.2) using Stampy (14), as described previously (4). Samtools (25) was used to assess sequencing and mapping quality: high-quality bases were considered to be those passing the −q30 and −Q30 30 filters (read quality and mapping quality all >30). Consensus sequence was called, requiring a minimum read depth of 5, including at least 1 read on each strand. Where an alternative base represented more than 10% of read depth, the base was recorded as uncertain, as described previously (26).

The variant call format (VCF) file was parsed with custom python scripts, and the number of high-quality bases (defined using the filters above) at each position was determined. These frequencies were extracted, stored, and indexed using SQLite with a python API constructed to allow extraction of mixture frequencies in arbitrary positions.

### Modeling minor variant frequencies.

We determined the most common (major) variant at each position. All other variants are considered minor variants. We define *n* as the total sequencing depth at one base, *m* as the depth of most common variant at one base, and *m*′ as the depth of all variants other than most common, *n* − *m* (see Fig. S1 in the supplemental material).

We divided up the H37Rv genome based on the annotation in NC_000962.3, identifying 8,007 regions R, comprising open reading frames and regions between open reading frames (Data Set S1 in the supplemental material).

For each of these regions *j* = 1.0.8007, if the region has length *l_j_*, the total number of minor variant bases *V_j_* across the i = 1…*l_j_* bases in the region is given by equation 1 and the total read depth *D_j_* across the gene by equation 2.

(1) $$V_{j}=\overset{l}{\underset{i=1}{\sum }}{m^{\prime }}_{l}$$

(2) $$D_{j}=\sum_{i=1}^{l}n_{l}$$

In order to describe the relationship between nonmycobacterial DNA quantifications and minor allele frequency, we stratified the number of reads from each sample identified as being from bacterial genera other than Mycobacterium (*b*) into four approximately equal-size strata: *b* < 1%, 1% ≤ *b* < 5%, 5% ≤ *b* < 20%, and *b* ≥ 20%.

We constructed separate Poisson regression models relating minor base counts (*V*) for each of the 8,007 regions [with log link and offset log(D)] to the nonmycobacterial bacterial read categories *b* (reference category < 1%), excluding any samples with zero high-quality depth in that region. We applied Bonferroni correction to model outputs to control for multiple testing (α = 0.01/8,007 = 1.2 × 10^−6^).

### Comparing impact of masking on pairwise comparisons.

Based on analysis of model output (see Results), regions with higher minor variant counts than expected were identified. These regions were excluded from pairwise comparisons performed using the findNeighbour2 tool (27).

### Impact of mapper.

For a random 250-sample subset, we compared the impacts of different mappers on minor variant frequencies across regions using the pipeline described above. We compared mapping with Stampy 1.0.32 (14), and Bowtie v 1.2.2 (11) with default parameters, and Bowtie2 v 2.3.4.1 with –very-sensitive and –very-fast preset parameters (28).

### Ethical framework.

Public health action taken as a result of notification and surveillance is one of the Public Health England's key roles as stated in the Health and Social Care Act 2012 and subsequent government directives which provide the mandate and legislative basis to undertake necessary follow-up. Part of this follow-up is identification of epidemiological and molecular links between cases. This work is part of service development carried out under this framework, and therefore, explicit ethical approval is unnecessary.

### Availability of data.

The identifiers for the samples studied are in the supplemental material, and the raw sequence data are in the NCBI under project number PRJNA302362. Software to effect the process described, test data, links to the data used, and documentation is freely available at https://github.com/davidhwyllie/adaptivemasking.

## RESULTS

### Samples studied.

**FIG 1:**
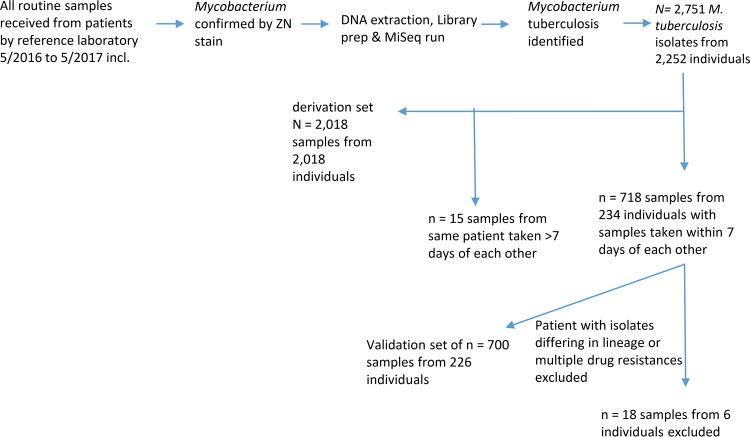
Samples used and derivation and validation sets. Shown is a flowchart describing the samples used and the selection of derivation and validation sets.

**FIG 2:**
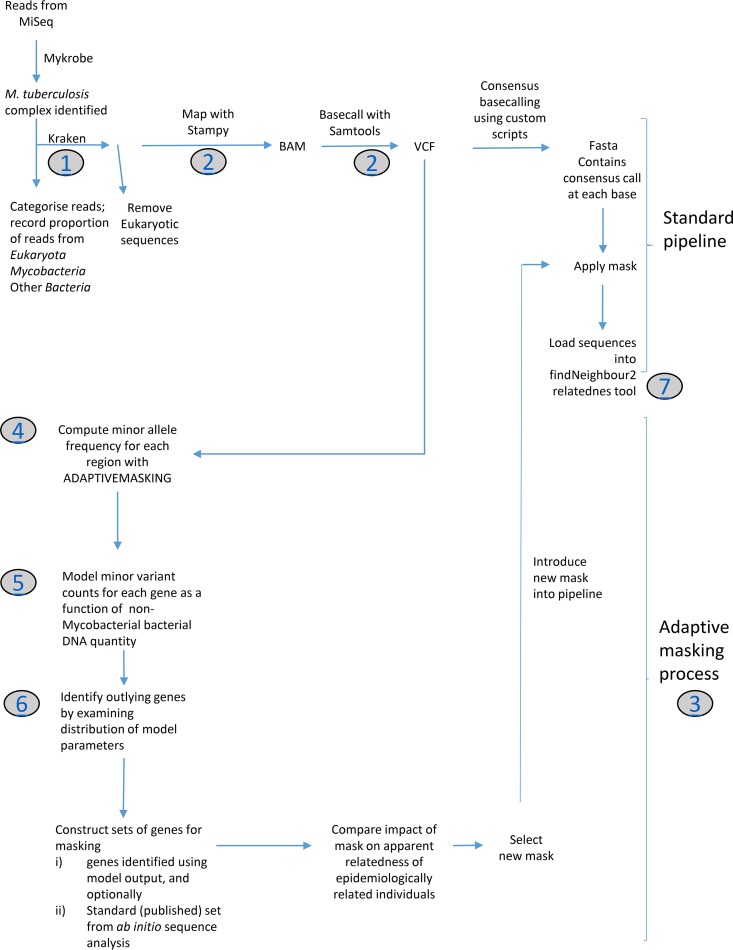
Bioinformatics processes. Shown is a flow diagram illustrating the standard bioinformatics pipeline used, as well as the adaptive-masking process used to generate masks. Gray circles indicate links to a description of the process at https://github.com/davidhwyllie/adaptivemasking. findNeighbour2 is an open-source server-based system for monitoring single nucleotide variation (27).

The PHE National Mycobacteriology Reference Laboratory Midlands implemented a laboratory process in which specimens received are decontaminated and inoculated into MGIT bottles, DNA extracts from positive MGIT bottles are made, and their contents are determined using Illumina short-read sequencing (17). Using this process, in the 13 months from 1 May 2016 to 30 May 2017, M. tuberculosis was identified in 2,751 samples sent from 2,252 patients (Fig. 1). Using these samples, we derived and validated using an independent validation set (Fig. 1) a strategy for investigating and controlling false-positive variation between samples, which we here term adaptive masking (Fig. 2). The initial stages of adaptive masking involve estimating minor variant frequencies across the genome from mapped data and determining whether these are related to the amount of nonmycobacterial DNA present.

We identified 718 samples from 234 individuals from whom more than one positive sample had been obtained with 7 days of another. Of these, for six individuals samples were reported as either being of different lineages, as defined previously (29), differing in multiple drug resistances, or differing by >400 high-quality SNVs. These observations we considered likely due to laboratory or sampling mix-ups, and samples from these patients were excluded. The other 700 samples were used as an independent validation set. From remaining samples, we identified the first sample from each of 2,018 individuals which were used to develop the adaptive-masking strategy (Fig. 1).

### Quantifying extraneous DNA and minor variant frequencies postmapping.

We determined the proportion of nonmycobacterial bacterial DNA in each sample using Kraken (23), mapped all reads to the H37Rv reference genome irrespective of Kraken results, and filtered the mapped data using stringent quality filters such that the expected error rate is less than 10^−3^ (see Materials and Methods). We defined 8,007 genomic regions in the reference genome; these regions comprise all canonical open reading frames and the genomic regions between them (Data Set S1). We were unable to assess one 15-nucleotide (nt) region between two PPE family members (positions 3380439 to 3380453), as no high-quality data mapped there in any sample.

**FIG 3:**
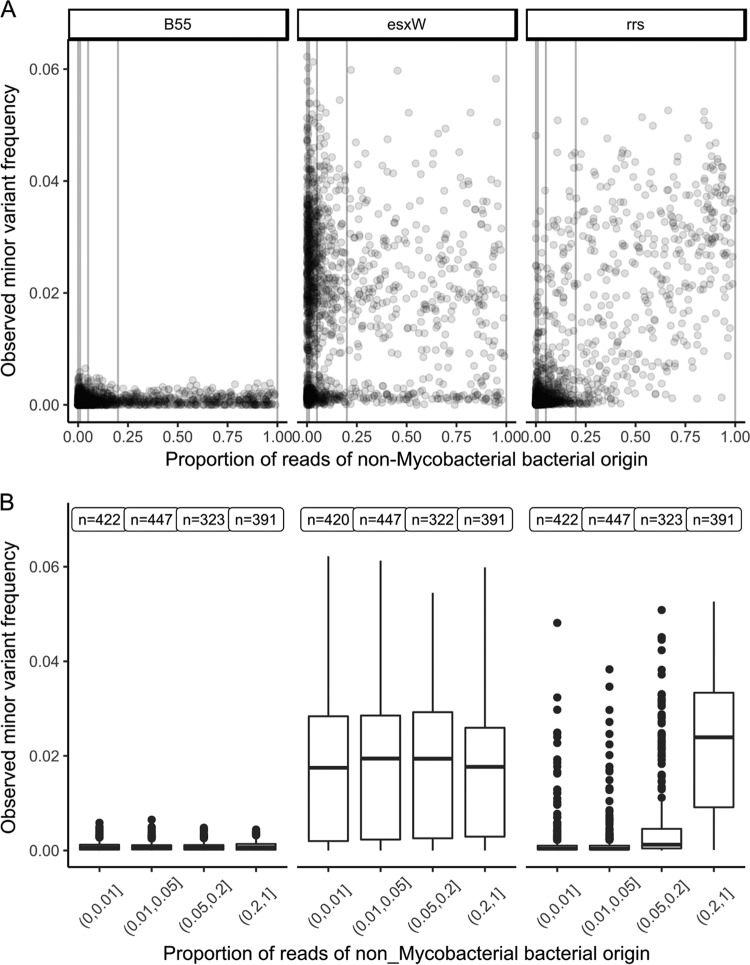
Minor variant frequency and nonmycobacterial bacterial DNA quantities. The observed minor variant frequency for three regions of the M. tuberculosis genome (genes *B55*, *eswX*, and *rrs*) versus the proportion of reads of nonmycobacterial bacterial origin (as determined by Kraken) is shown for samples in the derivation set (*n* = 2,018). Panel A shows a dot plot, whereas in panel B, the proportion of reads of nonmycobacterial bacterial origin is stratified with 1%, 5%, and 20% boundaries. The number at each stratum refers to the number of samples with nonzero read depth in that region.

In the other 8,006 regions, we observed that both minor variant frequencies and the relationship between minor variant frequency and the number of reads of nonmycobacterial origin differed markedly by gene. For example, the *B55* and *esxW* genes had, respectively, very low and very high minor variant frequencies, independent of nonmycobacterial DNA quantity. A small group of genes, of which the ribosomal component *rrs* is an example, showed low minor variant frequencies, except when nonmycobacterial DNA was present (Fig. 3).

### Estimating the impact of extraneous bacterial DNA.

**FIG 4:**
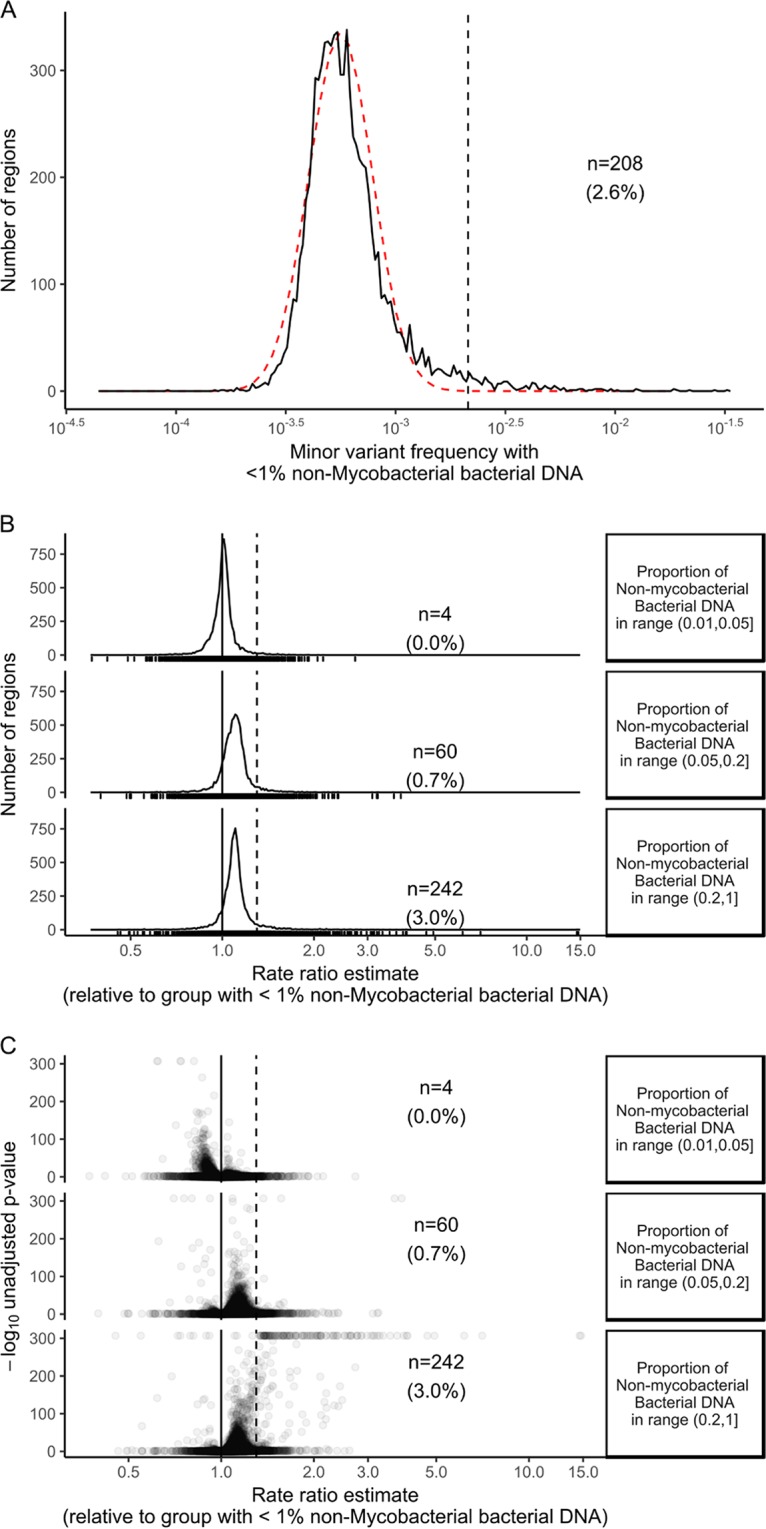
Modeling minor variant frequencies. For 8,006 genomic regions of the H37Rv reference genome, Poisson models were used to estimate the mean minor variant frequency. The estimated minor variant frequency when less than 1% nonmycobacterial bacterial DNA is present (*n* = 208 [2.6% of the regions]) is shown in panel A. The red line is a lognormal distribution with μ = log(minor variant frequency with <1% nonmycobacterial DNA) and σ = median absolute deviation [log(minor variant frequency with <1% nonmycobacterial DNA)]. In panel B the rate ratio estimates (i.e., the fold change associated with increases in nonmycobacterial bacterial DNA quantifications) for each gene are shown. Panel C shows the significance of a test comparing the log(rate ratio estimates) with zero, in the form of a Volcano plot. The dashed lines in panels B and C correspond to a 50% increase in rate ratio.

We modeled the relationship between minor variant counts and the number of nonmycobacterial reads, divided into four approximately equal-size strata (Fig. 3B), using Poisson models (Data Set S2). Separate models were constructed for each region. Estimated minor variant frequencies in samples with <1% nonmycobacterial bacterial reads had a median of 5 × 10^−4^ (Fig. 4A) across the 8,006 genomic regions, which is compatible with the expected mapping error rate of <10^−3^, given the filters applied.

The distribution of minor variant frequencies when less than 1% nonmycobacterial bacterial DNA was present approximated a lognormal distribution with mean log(5 × 10^−4^) and standard deviation equal to the median absolute deviation (Fig. 4A, observed, black line, and fitted, red line), but with a tail to the right. A total of 208 regions (2.6% of the total 8,006 regions), including *esxW* as well as other *esx* and PPE family members, had estimated minor variant frequencies of >2.1 × 10^−3^ when <1% nonmycobacterial bacterial reads were present (Fig. 4A; see also Data Set S2). This cutoff represents four median absolute deviations above the median; if the data were lognormally distributed, 24 samples would be expected with minor variant frequencies greater than this, versus the 208 observed.

Overall, estimated minor variant counts rose as nonmycobacterial DNA concentration rose, but for most regions the increase was small (Fig. 4B): the median fold change in minor variant counts in the presence of >20% nonmycobacterial DNA versus <1% nonmycobacterial DNA was 1.097 (i.e., a 9.7% increase; interquartile range, 5.4% to 14.0%). A total of 242 regions (3.0%) had statistically significant increases (Fig. 4B and C), more than 50%. Most of these regions had the highest minor variant counts when >20% nonmycobacterial DNA was present, although a small number had similar minor variant counts, in the 5 to 20% range and the >20% range (Fig. S2B).

### Mutually exclusive regions with increased minor variant frequency.

**FIG 5:**
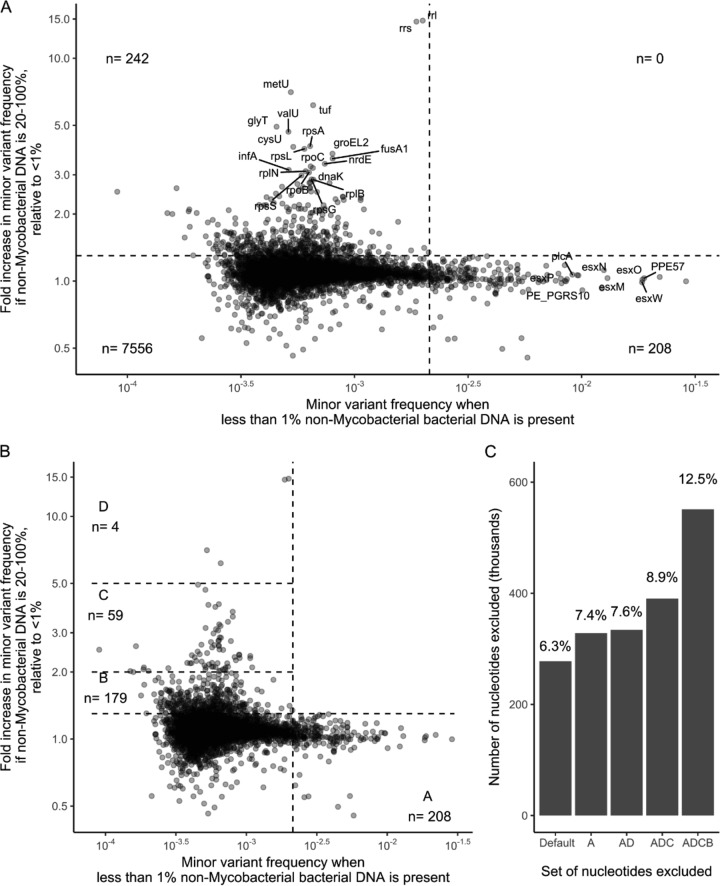
A distinct subset of genes are impacted by quantity of nonmycobacterial DNA. (A) Fold change in minor variant frequency with >20% nonmycobacterial bacterial DNA present versus <1% nonmycobacterial bacterial DNA. Quadrant boundary markers correspond to (horizontal line) a 50% increase over <1% nonmycobacterial bacterial DNA and (vertical line) a minor variant frequency of 2.1 × 10^−3^. (B) Genes with elevated minor variant frequencies when nonmycobacterial bacterial DNA is low (<1%) or high (>20%) fall into mutually exclusive sets. (C) The number of bases represented by the deployed masking versus the deployed masking plus the genes in zones A, A plus D, A plus D plus C, and A plus D plus C plus B.

Comparing regions with increased minor variant rates with low (<1%) nonmycobacterial bacterial DNA with those with increased minor variant rates with high (>20%) nonmycobacterial bacterial DNA shows these regions to be mutually exclusive (Fig. 5). The former include PPE and *esx* family members, while the latter include ribosomal components (*rrl*, *rrs*, *rplB*, and *rps* genes) as well as other highly conserved bacterial genes (tRNA genes, *fusA1*, *infA*, *dnaK*, and others) (Fig. 5; see also Data Set S2).

Examining Kraken read assignments in reads mapped to these highly conserved genes indicated that many reads mapping to these highly conserved regions cannot be unambiguously assigned to the M. tuberculosis taxon (Fig. S3) even when nonmycobacterial bacterial DNA is not present or is present in small amounts. For example, in the 422 samples for which <1% of total bacterial DNA is nonmycobacterial, only a small proportion (27.3%) of reads mapping with high quality to *rrs* are assigned to the Mycobacterium genus by Kraken. However, since very little, if any, nonmycobacterial bacterial DNA is present, these reads are almost certainly derived from M. tuberculosis rrs. In contrast, the corresponding figure for a gene with little homology with nonmycobacterial genomes (B55) is 97.4%. The corollary is that if one routinely removes reads which are not assigned by Kraken to the genus of interest (in this case Mycobacterium), one will remove a very high proportion of the reads corresponding to critical loci (including drug targets, such as *rrs*), even when no nonmycobacterial bacterial DNA is present, as occurs if one is sequencing pure cultures.

### Adaptive masking reduces the reporting of biologically implausible interindividual variation.

A published strategy for excluding regions of high mapping variation within the M. tuberculosis genome strategy masks (i.e., excludes from relatedness computations) 277,709 nt (6.3%) of the genome (4). Excluding regions with high estimated minor variant counts with <1% nonmycobacterial DNA (zone A in Fig. 5B) adds an additional 1.1%. Excluding regions with increased estimated minor variant counts only in the presence of >20% nonmycobacterial bacterial DNA (zones B to D) masks between 0.2% and 5.1% extra (Fig. 5B and C). The masking of regions identified by “adapting” to variation generated during the process forms the final part of the adaptive masking process.

**FIG 6:**
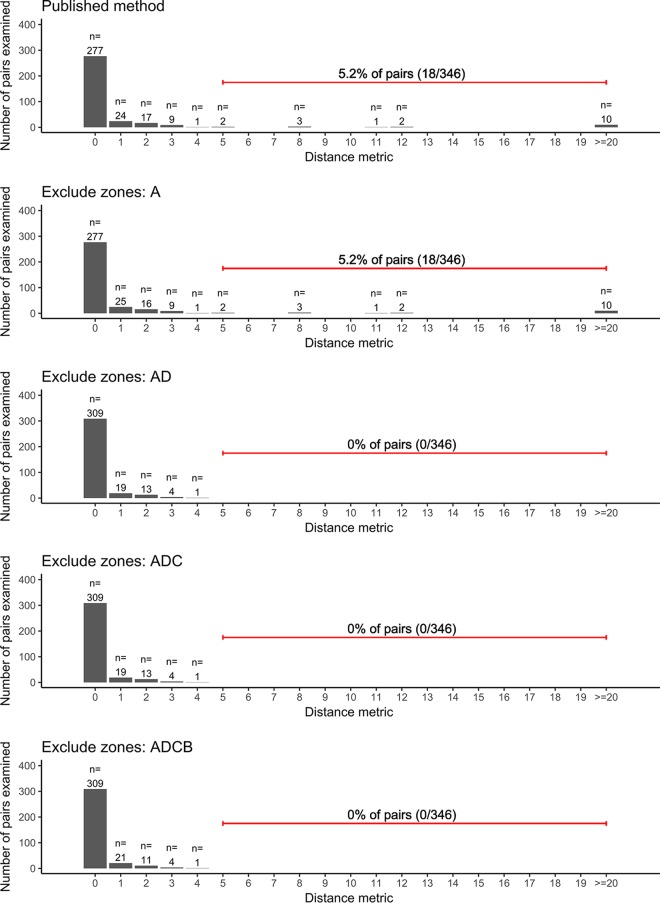
Impact of masking strategies on reported distances between closely related samples. SNV distances between pairs of M. tuberculosis genomes isolates from samples taken from the same individual within 7 days of each other were compared using different masking strategies. The top portion describes the published, deployed method of masking. In the panels below that, genes in the zones shown in Fig. 5B are additionally masked (i.e., ignored from pairwise comparisons).

In a validation set comprising isolates taken with 7 days of each other from 234 individuals, using the published strategy, 18/346 (5.2%) pairs studied had ≥5 SNVs; 10 of these had ≥20 SNVs. On exclusion of region D, which comprises the four genes most influenced by nonmycobacterial DNA, all encoding ribosome-associated products (the genes *rrl* and *rrs*, together with the tRNA *metU* and the highly conserved bacterial gene *tuf*), 0/346 pairs differed by ≥5 SNP (*P* <10^−4^ compared with the published method, Wilcoxon test on pairs). These genes were the only genes with minor variant frequencies significantly affected by nonmycobacterial DNA, i.e., at the 1 to 5% level (Fig. 4B, top portion; see also Data Set S2). Additional exclusion of genes in regions B and C, mapping to which is less influenced by nonmycobacterial DNA, had a limited impact (Fig. 6).

### Impact of mapper.

In the above-described work, we use the Stampy mapper (14) which forms part of the deployed TB bioinformatics pipeline (17). To determine whether the choice of mapper was important, we compared the results of mapping a 250-sample test set to the H37Rv reference genome using four different software/parameter settings. We identified regions with any significantly increased mixture frequencies when >20% nonmycobacterial bacterial DNA was present relative to when <1% was present using a *P* value of 0.05, adjusted by Bonferroni's method to 7.6 × 10^−6^. The numbers of such regions detected with Stampy, bowtie, and bowtie2 with stringent matching criteria and with bowtie2 with more relaxed matching criteria were 2,544, 25, 70, and 72, respectively (Data Set S3). Increases in minor variant frequencies in *rrl* were detected by all techniques; estimated fold increases in minor variant frequencies associated with >20% nonmycobacterial bacterial DNA were 37, 1.4, 1.5, and 1.5, respectively. Thus, although the mismapping observed identified occurred in all cases examined, it was much more prominent with Stampy than with the bowtie series of mappers.

## DISCUSSION

Here we describe an approach which we term adaptive masking. This involves monitoring the minor variant frequency across a bacterial genome to which sequencing reads have been mapped; consequently, it measures an end product of next-generation sequencing processes, taking into account the natural sequence variation in the samples studied as well as the impact of DNA extraction and library construction technologies and the performance of the mapping and filtering software used.

Using this approach, we defined a set of hard-to-map genetic regions (Fig. 5, zone A) with increased minor variant frequencies irrespective of the amount of nonmycobacterial bacterial DNA. Exclusion of these regions could be considered when assessing consensus M. tuberculosis sequences.

We also demonstrated a significant positive association between amounts of nonmycobacterial bacterial DNA and minor variant frequencies in a subset of the mapped genome: significant increases, of more than 1.5-fold, were observed in 242/8,006 regions examined, which together cover about 5% of the M. tuberculosis genome. Although the emphasis of this work was on relatedness between isolates, it is notable that included within the 242 regions are a series of genes encoding ribosomal components (*rrs*, *rrl*, *rpoB*, *rpsL*, and *rpsA*) which correspond to major antituberculosis drug resistance genes (26). Therefore, studies investigating resistance or heteroresistance using these loci should report estimates of the impact of the presence of nonmycobacterial bacterial DNA on heteroresistance estimates. Such interference may be particularly marked when direct-from-sample short-read sequencing is used (30), given the increased ratio of nonmycobacterial to mycobacterial DNA in the absence of selective mycobacterial amplification using culture.

Among these 242 regions, we identified four “high-variation” regions in which minor variant frequencies are very strongly influenced by nonmycobacterial bacterial DNA quantities, with fold increases in minor variant frequencies of >5 in the presence of >20% nonmycobacterial bacterial DNA. Importantly, if nonmycobacterial bacterial DNA concentrations are low (<1% of bacterial DNA present), as occurred in retrospective studies when mycobacteria were subcultured on Lowenstein-Jensen slopes prior to sequencing, increased variation is not observed in these regions. The exclusion of the four high-variation regions from base calling by a clinically deployed M. tuberculosis pipeline markedly reduced reported variation between samples derived from the same patient in a short period. In particular, prior to exclusion of the four high-variation regions, in a test set derived from 234 individuals, 5.2% of intrapatient pairs examined differed by 5 SNVs or more, with the majority of SNV differences observed in these pairs being >20. Multiple studies indicate that this is biologically implausible (3, 5, 7), and after exclusion of the four high-variation regions, comprising only 0.2% of the genome, no pairs had variation of 5 SNVs or more. This suggests that when using standard masking and DNA extraction from liquid media, false-positive variation is reported in a small number of sites in a nonmycobacterial bacterial DNA-dependent manner. Put alternatively, nonmycobacterial bacterial DNA acts as an interfering substance (20) for relatedness measurements.

A potential limitation of this work is that this approach studies prespecified regions of the genome, specifically coding regions and intergenic regions. This approach was chosen to avoid the challenges of analyzing the 4.4 × 10^6^ bases of the M. tuberculosis genome individually, with a concomitant loss of statistical power. Therefore, as described the method may neither detect nor allow selective masking of small regions with high minor variant frequencies within genes. A second limitation is that we did not use metagenomics classifiers, such as Kraken, to identify nonmycobacterial interfering DNA and eliminate it prior to mapping to the M. tuberculosis genome. We did not do this because we observed that for the highly conserved *rrs* genes, metagenomic classifiers cannot confidently assign reads to a genus level, likely because there is insufficient sequence variation within short-read sequencing of *rrs* to allow this. Therefore, until longer-read sequencing becomes available, sequencing less conserved flanking genomic regions, a strategy of read removal based on metagenomics classification, will eliminate a high proportion of bona fide M. tuberculosis-derived reads in conserved genes, even in samples without any nonmycobacterial bacterial DNA. Despite these limitations, the strategy chosen appears to be of use clinically, based on the reduction in likely false-positive variation between serial samples from individuals.

The routine clinical of use of next-generation sequencing is rapidly increasing (1, 4, 22). However, the reporting of microbial identity, resistotyping, and relatedness information requires complex, multistep processes whose outputs are dependent on specimen decolonization, selective culture, DNA extraction, library construction, DNA sequencing, and bioinformatics analysis (4). Reagent batches, software versions, and equipment involved in the process are all subject to change over time. The adaptive-masking approach we describe here represents a route to quantitative monitoring of the performance of the output of this pathway, identifying whether changes in process which may appear innocuous alter mapping and base calling across the genome. We do not propose that the output from the adaptive-masking process as demonstrated here with data generated by Public Health England and processed by particular bioinformatics tools should be used to generate a list of problematic genomic regions which can be universally applied. Rather, we envisage that the adaptive-masking process will be performed as part of the acceptance of process change, and periodically as part of quality monitoring, under the exact conditions used in the clinical laboratory issuing NGS-based results. A list of problematic positions to be ignored during relatedness calculations can then be fed into systems doing such calculations, such as findNeighbour2 (27), which would apply such masking across all samples.

Generalizable to other organisms and mapping pipelines, the adaptive-masking approach we describe here will have application in monitoring the performance of such processes quantitatively, in interpreting estimates of possible heteroresistance, and in preventing the calling of false-positive variation in the context of clinically deployed genomics.

## Supplementary Material

Supplemental material
