# Supplementary material for: Control of Artifactual Variation in Reported Intersample Relatedness during Clinical Use of a Mycobacterium tuberculosis Sequencing Pipeline
Source: J Clin Microbiol. 2018 Jul 26;56(8):e00104-18. doi: 10.1128/JCM.00104-18 (PMC6062814; doi:10.1128/JCM.00104-18)
Supplement: Supplemental material [file supp_56_8_e00104-18__index.html]

Supplemental material 

# Control of Artifactual Variation in Reported Intersample Relatedness during Clinical Use of a Mycobacterium tuberculosis Sequencing Pipeline

## Supplemental material

- Supplemental file 1 -

  Fig. S1 (Illustration of minor variant frequencies), S2 (Minor variant frequencies with increasing non-*Mycobacterium* bacterial DNA), and S3 (Identification of reads in selected regions by Kraken)

  PDF, 1.2M
- Supplemental file 2 -

  Data Sets S1 (Regions of H37Rv genome analyzed), S2 (Poisson model relating minor variant frequency to non-*Mycobacterium* bacterial DNA quantification), and S3 (Impact of different mappers)

  XLSX, 8.4M
