# Supplementary material for: Control of Artifactual Variation in Reported Intersample Relatedness during Clinical Use of a Mycobacterium tuberculosis Sequencing Pipeline
Source: J Clin Microbiol. 2018 Jul 26;56(8):e00104-18. doi: 10.1128/JCM.00104-18 (PMC6062814; doi:10.1128/JCM.00104-18)
Supplement: Supplemental material [file JCM.00104-18_zjm999096044s1.pdf]

Figure S1 Illustration of minor variant frequencies

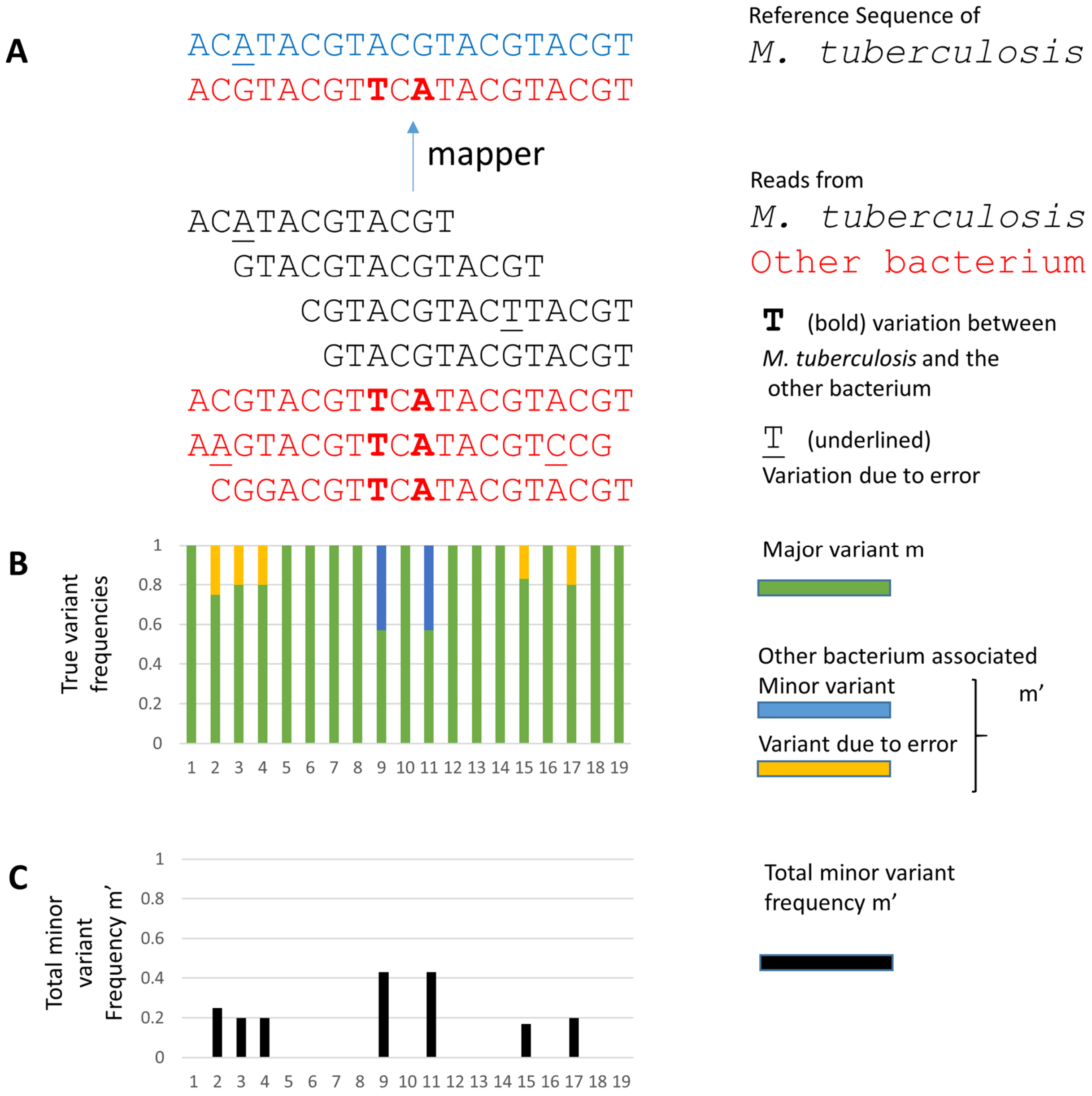

Legend: (A) Short read sequencing data, either from *M. tuberculosis* or from other bacteria (red) is mapped to a reference gene. Minor variants (B) can result either from sequencing error (underlined), or due to alignment of non-*Mycobacterium tuberculosis* DNA to the reference. Variation of both types contribute to the minor variant frequency (C).

Figure S2 Minor variant frequencies with increasing non-Mycobacterial bacterial DNA

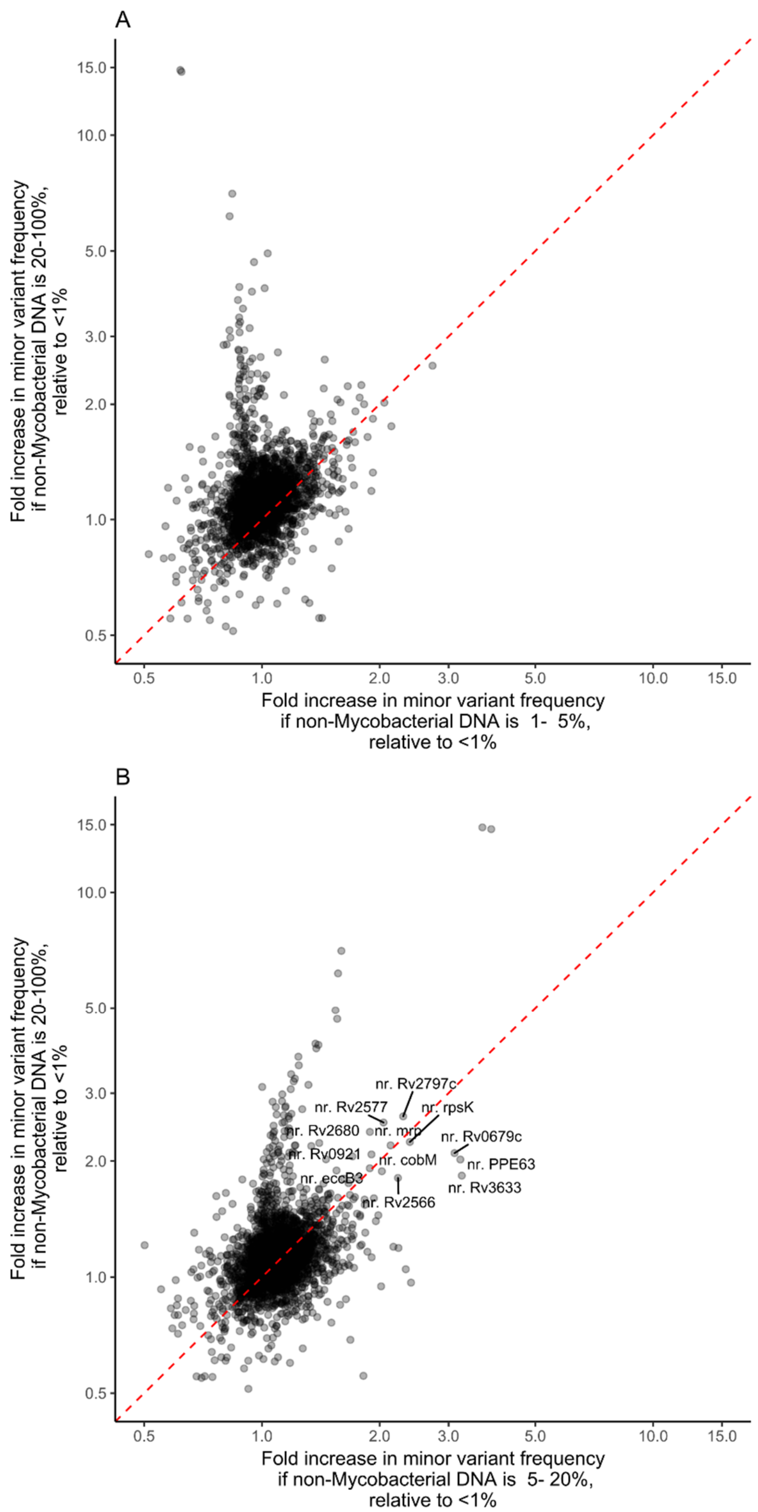

Legend: Estimated rate ratios derived from Poisson modelling of minor variant frequencies relative to frequencies when <1% non-Mycobacterial bacterial DNA was present. (A) compares rate ratios with 1-5% vs. >20% non-Mycobacterial bacterial DNA; (B) compares 5-20% vs. >20% non-Mycobacterial bacterial DNA. In (B), regions with similar rate ratios when 5-20% and >20% non-Mycobacterial bacterial DNA are annotated.

Figure S3 Identification of reads in selected regions by Kraken

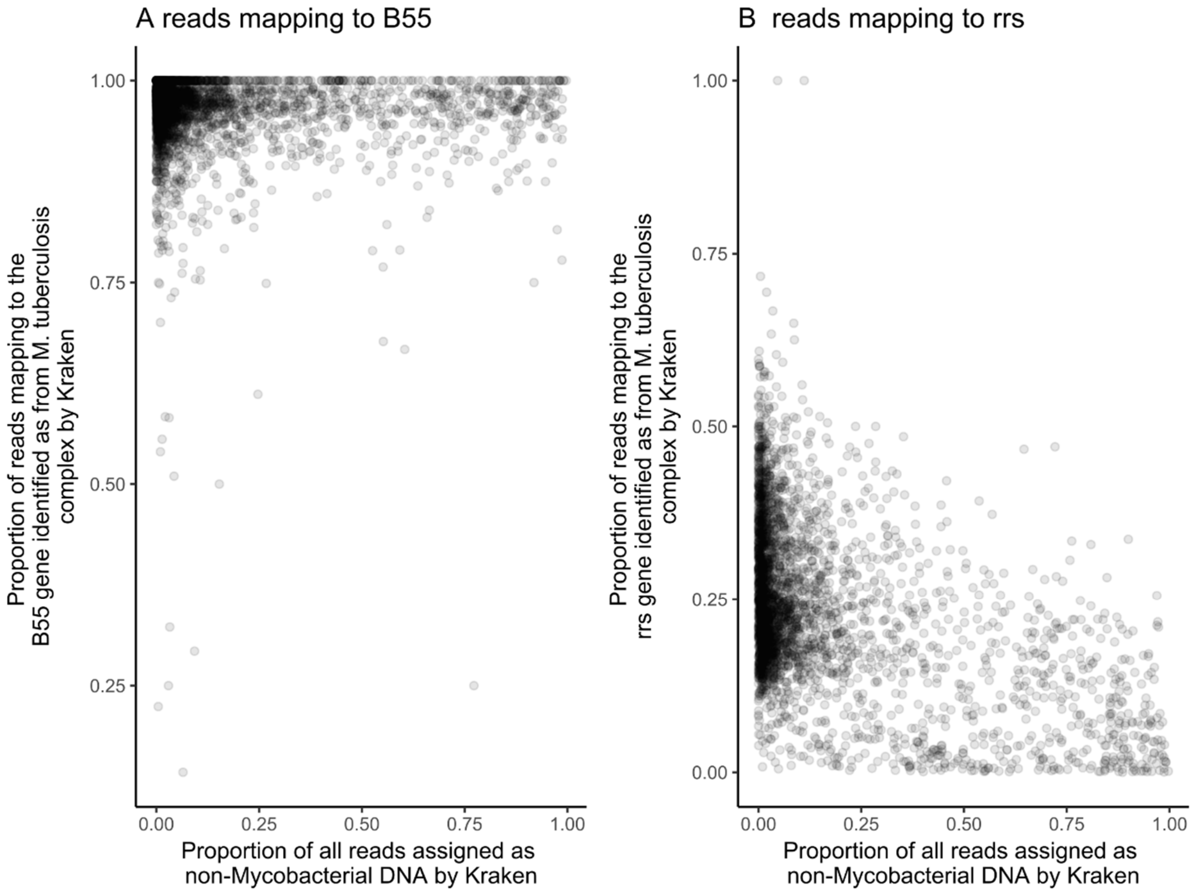

Legend: The proportion of reads mapped to (A) the B55 gene (B) the rrs genes which are identified as belonging to the *M. tuberculosis* complex by Kraken are shown, as is their relationship with the estimate of the amount of non-Mycobacterial bacterial DNA in the all reads.
